# Food consumption of workers in an information technology company: randomized clinical study

**DOI:** 10.47626/1679-4435-2023-1187

**Published:** 2024-11-14

**Authors:** Diana Souza Santos Vaz, Anne Cristine Rumiato, Inês Monteiro

**Affiliations:** 1 Enfermagem, Universidade Estadual de Campinas, Campinas, SP, Brazil; 2 Nutrição, Hospital Universitário de Londrina, Londrina, PR, Brazil

**Keywords:** occupational health, food and nutrition education, eating, saúde ocupacional, educação alimentar e nutricional, consumo alimentar

## Abstract

**Introduction:**

The structure of the workplace, work relationships, accessibility and quality of food and time for meals reflect on individuals’ eating habits.

**Objectives:**

Analyze the changes in the diet of workers in relation to the consumption of calories, macronutrients and micronutrients.

**Methods:**

Longitudinal study, lasting 8 months, randomized and descriptive clinical trial, with 95 information technology workers in the city of Campinas, state of São Paulo. The workers were divided into two groups based on a draw, the intervention group participated in monthly meetings and received messages via WhatsApp^®^ forwarded weekly, and the control group participated in two meetings. In the nutritional evaluation, the measurements of weight, height, waist circumference and skin folds were bicipital, tricipital, suprailiac and subscapular; they also responded to the 24-hour recall in which the consumption of macronutrients (protein, carbohydrates and lipids), micronutrients (vitamins A and C, calcium and selenium), calories and fiber was measured.

**Results:**

It was observed that there was a significant difference between the final and start consumption of protein (p = 0.0008), carbohydrate (p = 0.0053) and calcium (p = 0.0197) in the control and selenium group (p = 0.0049) of the intervention group.

**Conclusions:**

There were no significant changes in diet and in the reduction of body measures. The environment, time of intervention and frequency of individual consultations need to be reviewed for future studies, in order to improve commitment, make participation more active, for interventions to be effective.

## Abstract (pt)

**Introdução:**

A estrutura do local de trabalho, as relações de trabalho, a acessibilidade e qualidade dos alimentos e o tempo para as refeições refletem nos hábitos alimentares dos indivíduos.

**Objetivos:**

Analisar as mudanças ocorridas na alimentação dos trabalhadores em relação ao consumo de calorias, macronutrientes e micronutrientes.

**Métodos:**

Estudo longitudinal e ensaio clínico randomizado e descritivo, com duração de 8 meses, com 95 trabalhadores de empresa de tecnologia da informação do município de Campinas, estado de São Paulo. Os trabalhadores foram divididos em dois grupos a partir de sorteio; o grupo intervenção participou de encontros mensais e recebeu mensagens via WhatsApp^®^ encaminhadas semanalmente, e o grupo-controle participou de dois encontros. Na avaliação nutricional, foram aferidas as medidas de peso, estatura, circunferência da cintura e pregas cutâneas bicipital, tricipital, suprailíaca e subescapular. Também responderam ao Recordatório 24 horas, em que foi mensurado o consumo dos macronutrientes (proteína, carboidratos e lipídios), micronutrientes (vitamina A e C, cálcio e selênio), calorias e fibras.

**Resultados:**

Observou-se que houve diferença significativa entre o consumo final e inicial de proteína (p = 0,0008), carboidrato (p = 0,0053) e cálcio (p = 0,0197) do grupo-controle e selênio (p = 0,0049) do grupo intervenção.

**Conclusões:**

Não houve mudanças significativas na alimentação e na redução de medidas corporais. O ambiente, o tempo de intervenção e a frequência das consultas individuais precisam ser revistos para estudos futuros, no intuito de melhorar o comprometimento e tornar a participação mais ativa, para que as intervenções sejam efetivas.

## INTRODUCTION

Generally speaking, workers in Brazil work 40 hours per week and spend at least 50% of their day at work. The structure of workplaces, working relationships, accessibility and quality of food, and mealtimes all have an effect on eating habits, weight, lifestyle, and occupational risk factors, to name a few.^1^

The dietary pattern of Brazilian people has been changing over the years, which may be associated with the difficulty of defining their identity due to their own history, colonization, or their inability to find a place of origin, which makes importing, absorbing and incorporating new habits and abandoning their own traditions rather easily. In addition to the process of globalization, purchasing power, advertising, and convenience permeate changes such as the way food is purchased and consumed, the way meals are prepared, and the introduction and inclusion of new food. According to data reported by the Sistema de *Vigilância de Fatores de Risco e Proteção para doenças Crônicas por Inquérito Telefônico* (VIGITEL, Brazil Surveillance System of Risk and Protective Factors for Chronic Diseases via Telephone Survey),^2^ only 34.2% of adults ingest fruit and vegetables regularly (five times per week or more), 18.2% reported they had ingested five or more ultraprocessed food groups the day before the interview, in addition to swapping the traditional meal (rice, beans, meat, and salad) for fast food and ingesting larger servings of sugary food and drinks. These results could change once the focus of public policies and national health education is on eating healthily.^2^

According to the literature,^3,4^ eating healthily should be based on eating habits that take the social and cultural significance of food as their underlying, conceptual foundation. It is therefore essential to reclaim these traditions and encourage the production and ingestion of healthy local dishes such as legumes, vegetables, and fruit, which are packed with vitamins and minerals, and generally contain very few calories. They are therefore excellent options for preventing nutrient deficiencies and aiding in the process of curbing excessive calorie intake and obesity.

Macronutrients in the form of fat, protein, and carbohydrates provide energy and essential components for sustaining life. Fat is composed of glycerol and fatty acids; protein is an agglomeration of amino acids; and carbohydrates are simple sugars that occur as monosaccharides or chains of connected monosaccharides (e.g. starch) whose bonds are hydrolyzed in the human small intestine into monosaccharides or are resistant to hydrolysis (dietary fiber).^4^

A combination of these macronutrients in our diet is necessary to maintain longevity and health. It is questionable whether there is a combination of macronutrients that provides optimal health. When expressed as a percentage of energy in the diet, human populations have historically survived on diets with very different ratios of these macronutrients.^5^

In addition to macronutrients, dietary fiber, which plays an indispensable role in human health, is composed of carbohydrate polymers with three or more monomeric units, and lignin. The benefit of fiber is related to the fermentation process that occurs in the large intestine, reducing the speed of intestinal transit, which results in a lower pH in the colon, causing the environment to become more acidic. This prevents the formation of pathogens and facilitates the absorption of calcium, interfering with bone metabolism and reducing the solubility of bile acids.^6^

Accordingly, fiber is recommended at approximately 25 to 30 g/day for adults, as its benefits have been proven, especially in terms of reducing the risk of chronic noncommunicable diseases, coronary artery disease, stroke, hypertension, and diabetes, improving the lipid profile and some intestinal disorders, and having a positive relationship with the immune system and reduced body weight. It is therefore associated with health treatment and physical activity, a low-calorie diet, and medication.^6^

This study aimed to analyze how workers’ diets have changed in terms of calories, macronutrients, and micronutrients intake, given the various factors that influence the diet and nutritional status of adults.

## METHODS

This is a longitudinal study and a randomized, descriptive clinical trial. The sample consisted of employees from an information technology company, both men and women, from the municipality of Campinas, São Paulo, Brazil. Most of the employees worked sitting down in front of a computer.

The sample size assumed a significance level of 5%, with a test power of 80% and an effect size of 0.25, which can be considered a medium effect size.^7^ The sample size was 98 individuals (49 per group). The G* Power software version 3.1.9.2^7-9^ was used to calculate the sample.

We collected data over a period of 8 months (May to December 2020) at the outpatient clinic of the company. Employees who agreed to participate in the survey signed an informed consent form and were then drawn into two groups according to the buildings in which they worked (intervention group [IG]: buildings 2, 4, and 6; control group [CG]: buildings 3, 7, and 13), and one-on-one assessments were scheduled.

As part of their personal assessment, employees completed a 24-hour recall (R24h),^10^ which analyzed their intake of macronutrients (proteins, carbohydrates, and lipids), micronutrients (vitamins A and C, calcium, and selenium), and fiber; and nutritional assessment, in which weight, height, waist circumference (WC) and bicipital (BCP), tricipital (TCP), suprailiac (SCSi), and subscapular (SCSe) skinfolds were measured.^11^

Body mass index (BMI) was ranked according to the World Health Organization (WHO),^12^ where < 18.5 kg/m^2^ indicates underweight, ≥ 18.5 and < 24.9 kg/m^2^, adequate weight, ≥ 25 and < 29.9 kg/m^2^, overweight, ≥ 30 and < 34.9 kg/m^2^, class I obesity, ≥ 35 and < 39.9 kg/m^2^, class II obesity, and ≥ 40 kg/m^2^, class III obesity. In this study, participants were divided into two types: underweight/adequate weight and overweight/obesity I, II, or III.^11^

WC is a risk indicator when it is greater than 94 cm for men and 80 cm for women, with values below 80 cm being adequate for women and below 94 cm for men and values above these figures are considered high.^11^

Body fat (BF) was ranked according to Pollock & Wilmore,^13^ where high refers to obesity and normal/average corresponds to adequate/good. At the end of the consultation, participants were informed of their nutritional assessment results, which were also sent by e-mail (Chart 1).

**Chart 1:** Classification of BF of men and women, Campinas, SP, Brazil

Classification	BF	
	Men	Women
Malnutrition	< 6	< 8
Normal	6-24	9-31
Average	15	23
Obesity	> 25	> 32

Source: Pollock & Wilmore.^13^

BF = body fat.

The IG attended monthly meetings, based on the Guia Alimentar para a População Brasileira^4^ (Food Guide for the Brazilian Population) and weekly messages were sent via WhatsApp^®^ with eating tips.^14,15^ Among the features frequently used on WhatsApp^®^ are one-on-one or group chats which allow users to communicate and share information. It is free of charge and accessible to most of the public.^14^ The CG participated in two meetings on labeling and food, health, and work.

In the statistical analysis, the unpaired Student’s *t*-test or the Mann-Whitney test was used to compare the quantitative variables between the groups, depending on data distribution. Data distribution was assessed using the Shapiro-Wilk test^16^ and the chi-squared test was used to study associations between qualitative variables and groups.^16,17^ When the assumptions of the chi-squared test were not met, Fisher’s exact test was used.^17^ SAS^®^ version 9.4 was used for the analysis.

The study was published in the Registro Brasileiro de Ensaios Clínicos (ReBEC, Brazilian Registry of Clinical Trials) with the number RBR-6h6ykrc.

The study complied with the requirements of Resolution No. 466/12 of the Conselho Nacional de Saúde (Brazil National Health Council), and the Research Ethics Committee approved the study with Certificado de Apresentação de Apreciação Ética (Certificate of Submission for Ethical Appraisal) No. 93132318.2.0000.5404.

## RESULTS

Both the IG and CG activities were held at the company during working hours. During field observation, several sites in the company premises were found offering chocolates, ice cream, milkshakes, filled cakes, açaí with powdered milk, condensed milk, and Nutella^®^, desserts such as flans, mousses, lemon tarts, *Brigadeiro* (Brazilian chocolate fudge balls), *Beijinho* (Brazilian coconut balls), *Casadinho* (Brazilian guava cookies), and others.

The descriptive analysis of the variables sex, age, BMI, WC, and BF at the beginning and end of the nutritional assessment showed that most of the participants were women (58.34%), aged between 19 and 60. The majority of participants in the IG were aged up to 29 and the majority of participants in the CG were aged between 40 and 49. In both groups, most of the workers were overweight and obese, according to the BMI classification, and had high BF (Table 1).

**Table 1:** Descriptive variables such as age and BMI, WC, and BF of information technology employees, according to time (n = 95), Campinas, SP, Brazil, 2020

Variable	Groups			
	IG		CG	
	n	%	n	%
Sex				
Male	19	37.25	20	45.45
Female	32	62.75	24	54.55
Age (years)				
< 30	18	35.29	7	15.90
30 to 39	15	29.41	13	29.54
40 to 49	15	29.41	16	36.36
≥ 50	3	5.88	8	18.18
BMI class. - T1				
Underweight/adequate	17	33.33	14	31.82
Overweight/obese	34	66.67	30	68.18
BMI class. - T2				
Underweight/adequate	18	35.29	15	34.09
Overweight/obese	33	64.71	29	65.91
WC class. - T1				
Adequate	26	50.98	20	45.45
High	25	49.02	24	54.55
WC class. - T2				
Adequate	26	50.98	20	45.45
High	25	49.02	24	54.55
Classification of BF - T1				
Adequate/good	5	9.80	7	15.91
High	46	90.20	37	84.09
Classification of BF - T2				
Adequate/good	3	5.88	7	15.91
High	48	94.12	37	84.09

BF = body fat; WC = waist circumference; CG = control group; IG = intervention group; BMI = body mass index; T1 = start time; T2 = end time.

A significant difference was observed between the final and start intake of protein (p = 0.0008) and carbohydrate (p = 0.0053) in the CG, with protein intake increasing and carbohydrate intake decreasing (Table 2).

**Table 2:** Dietary intake of macronutrients, fiber, and calories/weight of information technology employees according to IG (n = 51), CG (n = 44), and time, Campinas, SP, Brazil, 2020

Dependent variable	Comparison	Mean difference	95%CI		p-value
			Lower threshold	Upper threshold	
Proteins	IG-CG (T1)	3.11	0.28	5.94	0.0314
	IG-CG (T2)	0.30	-2.89	3.48	0.8553
	T2-T1 (CG)	3.88	1.62	6.13	0.0008
	T2-T1 (IG)	1.06	-1.87	4.00	0.4778
Carbohydrates	IG-CG (T1)	-4.44	-8.43	-0.45	0.0292
	IG-CG (T2)	0.21	-4.25	4.67	0.9271
	T2-T1 (CG)	-5.49	-9.35	-1.63	0.0053
	T2-T1 (IG)	-0.84	-4.05	2.36	0.6053
Lipids	IG-CG (T1)	1.33	-2.12	4.79	0.4502
	IG-CG (T2)	-0.52	-4.12	3.08	0.7762
	T2-T1 (CG)	1.62	-1.52	4.75	0.3117
	T2-T1 (IG)	-0.24	-3.10	2.63	0.8708
Fibers	IG-CG (T1)	1.70	-1.16	4.55	0.2441
	IG-CG (T2)	-0.25	-2.29	1.79	0.8085
	T2-T1 (CG)	0.35	-1.31	2.02	0.6772
	T2-T1 (IG)	-1.59	-3.71	0.52	0.1396
Calories per weight	IG-CG (T1)	2.84	-1.11	6.79	0.1587
	IG-CG (T2)	-0.75	-4.64	3.13	0.7039
	T2-T1 (CG)	2.24	-0.60	5.08	0.1228
	T2-T1 (IG)	-1.35	-3.93	1.22	0.3035

CG = control group; IG = intervention group; T1 = start time; T2 = end time; 95%CI = 95% confidence interval.

* Generalized estimating equations (GEE).

The main micronutrients and minerals are shown in Table 3. When calcium was analyzed in the CG, a statistical difference (p = 0.0197) was observed between T2 and T1. In the IG, selenium showed a statistical difference (p = 0.0049) between T2 and T1.

**Table 3:** Dietary intake of vitamins and minerals of information technology employees in the IG (n = 51), CG (n = 44), and time, Campinas, SP, Brazil, 2020

Dependent variable	Comparison	Mean difference	95%CI		p-value
			Lower threshold	Upper threshold	
Vitamin A	IG-CG (T1)	-138.48	-386.53	109.58	0.2739
	IG-CG (T2)	-156.57	-432.38	119.25	0.2659
	T2-T1 (CG)	99.40	-154.33	353.14	0.4426
	T2-T1 (IG)	81.32	-84.36	246.99	0.3361
Vitamin C	IG-CG (T1)	-18.69	-64.60	27.22	0.4249
	IG-CG (T2)	-21.02	-82.70	40.66	0.5042
	T2-T1 (CG)	25.73	-36.81	88.26	0.4201
	T2-T1 (IG)	23.40	-16.94	63.73	0.2556
Calcium	IG-CG (T1)	65.64	-43.91	175.19	0.2402
	IG-CG (T2)	-114.30	-347.29	118.68	0.3363
	T2-T1 (CG)	260.13	41.51	478.75	0.0197
	T2-T1 (IG)	80.18	-55.63	216.00	0.2472
Selenium	IG-CG (T1)	-6.22	-20.52	8.09	0.3945
	IG-CG (T2)	-7.23	-31.22	16.76	0.5546
	T2-T1 (CG)	17.61	-5.74	40.96	0.1394
	T2-T1 (IG)	16.59	5.04	28.14	0.0049

CG = control group; IG = intervention group; T1 = start time; T2 = end time; 95%CI = 95% confidence interval.

* Generalized estimating equations (GEE).

From the R24h, we divided food into groups according to the Health Eating Index (HEI).^18^ These groups are: whole fruit (fruit only), total fruit (includes whole fruit and fruit juice), total vegetables (all types of vegetable), green and yellow vegetables, total carbohydrates (cereals, roots, and tubers), whole carbohydrates, refined carbohydrates, dairy (milk and dairy products, soy-based drinks), proteins, oils (monounsaturated and polyunsaturated fats), and trans-fat food, alcohol, sugar, i.e. empty calories (GordAA).^18^

In the IG, when comparing the end time (T2) with the start time (T1), the intake of servings of whole fruit, total fruit, green and yellow vegetables, whole carbohydrates, proteins, and GordAA increased. However, intake of total vegetables, total carbohydrates, refined carbohydrates, dairy products, and oils decreased (Table 4).

**Table 4:** Descriptive of the mean number of food servings obtained in the R24h according to HEI^*^ (n = 95), Campinas, SP, Brazil, 2020

Food group	No. of servings			
	IG-T1	IG-T2	CG-T1	CG-T2
Whole fruit	1.06	1.25	1.55	1.43
Total fruit	1.41	1.51	1.93	1.90
Total vegetables	2.17	2.10	2.00	2.36
Green and yellow vegetables	1.02	1.26	1.17	1.47
Total carbohydrates	4.68	4.40	4.41	4.10
Whole carbohydrates	1.04	1.27	1.23	1.16
Refined carbohydrates	3.64	3.13	3.20	2.94
Dairy products	1.24	1.19	1.20	1.74
Proteins	1.87	2.06	1.93	2.40
Oils	1.03	1.02	1.07	1.07
GordAA	1.33	1.50	1.51	1.75

CG = control group; IG = intervention group; GordAA = food containing trans fats, alcohol, sugar - empty calories; R24h = 24-hour recall; T1 = start time; T2 = end time.

* Health Eating Index (HEI).

In the CG, when comparing T2 and T1, the intake of whole fruit, total fruit, total carbohydrates, whole carbohydrates, and refined carbohydrates decreased. However, the intake of total vegetables, green and yellow vegetables, dairy products, proteins, and GordAA increased (Table 4).

## DISCUSSION

In the IG, protein intake decreased and selenium intake increased, while in the CG protein and calcium intake increased and carbohydrate intake decreased. However, the results show that no statistically significant difference was found between the IG and CG with regard to diet and body measurements.

BMI, WC, and BF were above the recommended levels in both groups. The study was conducted in a large information technology company with sedentary working conditions.

Several factors can be related to high body fat levels, such as the type of work performed, the obesogenic environment, psychological, physiological and genetic issues, and the supply/food intake - especially ultraprocessed food. The Estudo Longitudinal de Saúde do Adulto^19^ (ELSA-Brazil, Longitudinal Study of Adult Health) assessed the association between intake of ultraprocessed food and weight gain and increased WC, and concluded that high intake of ultraprocessed food may contribute to the obesity epidemic in Brazil and worldwide.

Protein is a macronutrient whose intake is encouraged and discussed by professionals who treat overweight and obese individuals.^20^ Animal and plant-based protein plays an important role in weight loss and reducing BF, since it increases satiety, reduces postprandial hunger, and helps to reduce WC, blood pressure, plasma levels of fat and sugar, and helps to build muscle fibers when ingested in combination with physical activity.^20^

Investigators^21^ have shown the benefits of protein intake, especially vegetable protein, which is associated with lowering the risk of chronic noncommunicable diseases such as hypertension, cardiovascular disease, dyslipidemia and diabetes, and reducing WC, weight, and BF. Although the relationship between protein and mortality has not been investigated, Naghshi et al.^21^ found that adequate intake of total protein has an inverse association with mortality. The addition of 3% vegetable protein per day corresponds to a 5% lower risk of death from various causes. This corroborates previous discussions on the benefits of protein, particularly plant-based protein, which can be found in beans, chickpeas, lentils, peas, and soybeans, for example.^21^

Carbohydrates are rich in fiber, vitamins and minerals and help with intestinal motility, intestinal microbiota activity, blood glucose maintenance, and are also a source of energy for the brain, nervous system, red blood cells and other tissues, in addition to other physiological functions such as weight maintenance and weight loss due to their high fiber concentration and feeling of satiety.^22^ Liu et al.^22^ also showed the importance of carbohydrate intake and the prevention of diseases such as type-2 diabetes and colorectal cancer, since complex carbohydrates, especially whole grains, have antioxidant properties and are a source of fiber and vitamins.^22^

According to the division of food into groups proposed by the HEI,^18^ the CG increased their intake of total vegetables, green and yellow vegetables, dairy products, proteins, and GordAA. Increased intake of calcium in the CG may be related to increased intake of dairy products, since they are a source of this micronutrient. The IG increased the intake of whole fruit, total fruit, green and yellow vegetables, whole carbohydrates, proteins, and GordAA, and increased intake of selenium-source food.^18^

Just as some carbohydrates have antioxidant properties, so does selenium, not to mention its anti-inflammatory activity, control of thyroid hormones, benefits for the immune system, cancer prevention, muscle health, and regulation of hormones and glucose metabolism. Selenium can be found in plant and animal-based food, including seafood, meat, poultry, eggs, dairy products, bread, cereals, and other grains such as Brazil nuts.^23,24^ The nutritional properties of selenium may be positively related to the performance of these employees, reducing absenteeism by preventing and reducing illnesses as it provides better immunity and muscle health, which is very positive for the health of adult individuals.^18^

Another important point is that most of the food ingested had a good outcome in terms of body measurements and health benefits. This shows that educational, communication, and information actions included in the Estratégia Intersetorial de Prevenção e Controle da Obesidade (Brazil Intersectoral Strategy for the Prevention and Control of Obesity) has had a positive effect on this population.^6^

A study^25^ that explored potential barriers and facilitators to healthy eating in consumers’ food environment, when analyzing 573 stores, found a high availability of ultraprocessed food with high energy density and low nutritional density, such as sugary drinks, candies, chocolate, cookies, corn snacks, and ice cream, especially in supermarkets, local markets, bakeries, and butchers.

Hobbs et al.^26^ found that an obesogenic environment has automatic and unconscious influence on behavior due to the opportunities and living conditions that promote obesity in individuals and populations, a characteristic that was very present in the environment of the company in which the study was conducted. Both the accessibility and supply of ultraprocessed food can influence the nutritional profile of employees; therefore, these issues should be revised so that actions to promote health and quality of life are effective.^27^

Thus, health education in the workplace is important, although challenging due to the high workload and work demands in some companies.^28^ Gea Cabrera et al.^29^ also state that health interventions in the workplace are essential for improving workers’ health and well-being. A study^28^ with a multidisciplinary team that provided physical activity and nutritional consultations for 302 health professionals at a Unidade Básica de Saúde (UBS, Primary Health Care center) concluded that BMI, BF, WC, waist-to-hip ratio, and waist-to-height ratio of employees were effectively improved, regardless of sex, age, and weight.

This study has limitations including the CG consisting of older individuals, which provides maturity and greater concern for health, the obesogenic environment, participants missing the proposed meetings due to work responsibilities, contact between workers in the IG and CG due to the change of building and sectors that occurred during the study, and the loss of the sample due to redundancies in the company. According to nutrition randomized clinical trials, nutritional interventions have more effective results if they last one year or more.

## CONCLUSIONS

We concluded that in the IG selenium intake increased and protein intake decreased, while in the CG protein and calcium intake increased and carbohydrate intake decreased. However, no significant changes in diet or reducing body measurements were observed. The environment, intervention time, and frequency of individual consultations should be reviewed in future studies so as to improve commitment and increase active participation to ensure that the interventions are effective.

Nutrition education based on scientific evidence can lead to more conscious and responsible food intake, prioritizing sustainable food choices, avoiding waste, and focusing on the food intake as an ally for health, quality of life, and environmental protection.
